# How to update esophageal masses imaging using literature review (MRI and CT features)

**DOI:** 10.1186/s13244-024-01754-0

**Published:** 2024-07-06

**Authors:** Jinrong Qu, Zhaoqi Wang, Hongkai Zhang, Yanan Lu, Zhengyan Jia, Shuang Lu, Keke Zhao, Funing Chu, Bingmei Bai, Yan Zheng, Qingxin Xia, Xu Li, Shaoyu Wang, Ihab R. Kamel

**Affiliations:** 1 Department of Radiology, the Affiliated Cancer Hospital of Zhengzhou University & Henan Cancer Hospital, Zhengzhou, Henan 450008 China; 2 Department of Thoracic surgery, the Affiliated Cancer Hospital of Zhengzhou University & Henan Cancer Hospital, Zhengzhou, Henan 450008 China; 3 Department of Pathology, the Affiliated Cancer Hospital of Zhengzhou University & Henan Cancer Hospital, Zhengzhou, Henan 450008 China; 4 MR Scientific Marketing, Siemens Healthineers, Shanghai, 201318 China; 5 Department of Radiology, Johns Hopkins University School of Medicine, Baltimore, MD 21205-2196 USA

**Keywords:** Esophageal neoplasms, Magnetic resonance imaging, Computed tomography

## Abstract

**Abstract:**

MRI offers new opportunities for detailed visualization of the different layers of the esophageal wall, as well as early detection and accurate characterization of esophageal lesions. Staging of esophageal tumors including extramural extent of disease, and status of the adjacent organ can also be performed by MRI with higher accuracy compared to other imaging modalities including CT and esophageal endoscopy. Although MDCT appears to be the primary imaging modality that is indicated for preoperative staging of esophageal cancer to assess tumor resectability, MDCT is considered less accurate in T staging. This review aims to update radiologists about emerging imaging techniques and the imaging features of various esophageal masses, emphasizing the imaging features that differentiate between esophageal masses, demonstrating the critical role of MRI in esophageal masses.

**Critical relevance statement:**

MRI features may help differentiate mucosal high-grade neoplasia from early invasive squamous cell cancer of the esophagus, also esophageal GISTs from leiomyomas, and esophageal malignant melanoma has typical MR features.

**Key Points:**

- MRI can accurately visualize different layers of the esophagus potentially has a role in T staging.
- MR may accurately delineate esophageal fistulae, especially small mediastinal fistulae.
- MRI features of various esophageal masses are helpful in the differentiation.

**Graphical Abstract:**

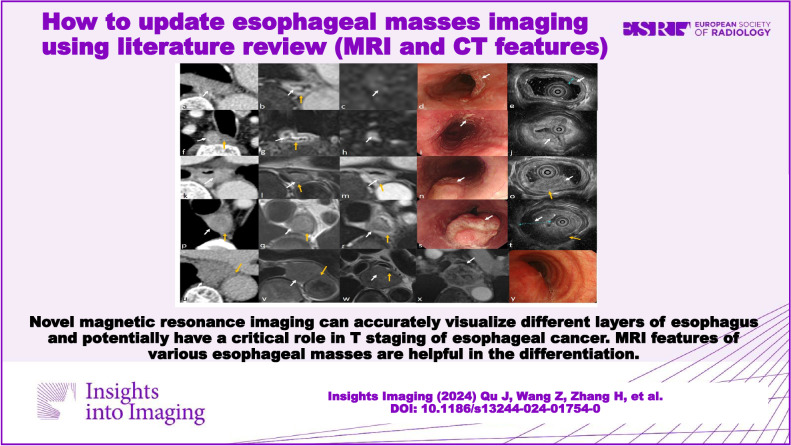

## Introduction

Novel MRI techniques offer high-resolution imaging of the esophagus [1], and have overcome the shortcomings of conventional MRI sequences in the chest [2]. MRI is a valuable tool for the evaluation of esophageal wall disease and serves as an adjunct to endoscopy. It enables high-resolution scanning of the entire esophagus using motion-insensitive sequences. [3]. In contrast to CT and endoscopy, MRI provides detailed information about the esophageal wall as well as the extramural extent of the disease. This is especially important for assessing the potential involvement of adjacent organs [4]. Familiarity with the imaging features of rare esophageal masses is also important. The purpose of our study is to familiarize radiologists with relatively new imaging techniques, which are significantly less motion-sensitive compared to conventional imaging techniques. Imaging features of various esophageal masses will be discussed, emphasizing the imaging features that differentiate between various esophageal masses, shown in Table 1.

**Table 1:** Key features of esophageal masses

Esophageal mass and mass-like conditions	Most common location	Boundary	Enhancement pattern on contrast-enhanced T1WI and CT	Special signs
Malignant				
EC	Middle esophagus	Ill-defined	Mild	Unintact mucosa on contrast-enhanced T1WI
ENEC	Distal end	Well-defined	Heterogeneous enhancement and moderate to markedly intense enhancement	Unintact mucosa on contrast-enhanced T1WI Central necrosis with high signal on T2WI and low signal on T1WI
Carcinosarcoma	Middle thoracic or lower	Ill-defined	Moderate inhomogeneous	Osseous component with low signal on both T1WI and T2WI only invade the layer of muscularis propria on both T2WI and contrast-enhanced T1WI without invading nearby organs
Lymphoma	Entire esophagus	Sharply delineated or irregular borders or well or ill-defined		Uniform restriction on DWI
Malignant melanoma	Middle or lower thoracic	Well-defined	Homogeneously varying degrees of enhancement	Uniform restriction on DWI
Benign				
Leiomyoma	Proximal end	Well-defined	Slightly homogenous	Intact mucosa on contrast-enhanced T1WI No restriction on DWI Young male Intramural eccentric on both T2WI and contrast-enhanced T1WI
Schwannoma	Middle or upper thoracic	Well-defined (benign); ill-defined (malignant)	Heterogeneity	Soft esophageal wall with no luminal obstruction Intact mucosa on contrast-enhanced T1WI
Lipoma	Entire	Well-defined	Unenhanced	Fat component with low intensity on CT or high signal on both T1WI and T2WI, drop in signal after fat suppression
Hemangioma	Cervical	Ill-defined	Submucosal homogeneous strong or gradually enhancing mass	Pedunculated on both T1WI and T2WI Nodular calcifications with high intensity on CT Peripheral puddling of contrast medium on contrast-enhanced T1WI
Fungal esophagitis	Entire esophagus	Well-defined	Unenhanced	Foamy appearance on double-contrast esophagography Feather appearance on double-contrast esophagography
Benign or malignant				
GISTs	Distal end	Well-defined	Heterogeneous	Intact mucosa on contrast-enhanced T1WI Restriction on DWI Larger (> 10 cm) Intramural eccentric on both T2WI and contrast-enhanced T1WI

### MR imaging techniques

A suggested MR imaging technique is shown in Table 2, patients are positioned head-first supine and the esophageal transverse plan was preferred for the localizer. To reduce esophageal peristalsis, raceanisodamine hydrochloride is injected intramuscularly 15–20 min before MRI. 3-T MRI with a diaphragm navigation T2 weighted turbo spin-echo (T2W TSE) sequence, diffusion-weighted imaging (DWI), and a 3D-GRE after contrast injection with free breathing show higher accuracy of staging in preoperative T staging of esophageal cancer [5] and assessing response to neoadjuvant chemotherapy [6]. MR image quality is significantly correlated with the use of coils, and a phased-array coil with as many channels as possible is recommended—with no fewer than 16 channels. The phased-array coil placement is basically in line with the esophagus, with the lesion generally placed in the center of the coil (a combined head and neck coil should be added to cover the upper mediastinum in patients with lower esophageal lesions), moving the examined area to the center of the magnetic field. Diffusion-weighted sequence scans were performed to demonstrate the location of the lesion first, and in the case of Siemens machines, sagittal and coronal diffusion-weighted images were reconstructed after feasible axial DWI, sequentially scanning axial T2 FSE fat suppression images, axial T2W TSE images with free breathing, and T1W images, followed by completion of axial enhanced images, sagittal and coronal enhanced images, and then post-enhanced axial T1WI scans of the cervicothoracic segment. For small lesions, it is valuable to acquire 1 mm-isotropic-3D contrast-enhanced 3D-GRE through the lesion [7].

**Table 2:** Key technical parameters of esophageal MR sequence

Sequence		Plane	Breathing control	FOV (cm)	TE (ms)	Section thickness (mm)	Matrix	NEX/fat suppression	Frequency direction	Pixel bandwidth Hz/pixel
1	DWI (SE-EPI)	TRA	Free breathing	34	55	5	128 × 96	2/Fat suppressed	A/P	2442
2	T2 FSE	TRA	Gate control	36	90–100	5	384 × 224	2/Fat suppressed	A/P	620
3	T1 3D	TRA	Breath holding	36	0.9	3	384 × 307	1	A/P	660
4	T2W TSE	TRA	Trigger	28	110	3	256 × 256	1/Non-fat suppression	A//P	710
5	Contrast-enhanced T1WI (3D-GRE)	TRA	Free breathing	38	MIN	3	288 × 288	1/Fat suppression	A//P	490
6	Contrast-enhanced T1WI (3D-GRE)	TRA	Free breathing	32	MIN	1	320 × 320	1/Fat suppression	A//P	490

Gadolinium-DTPA was injected at 0.1 mmol/kg through the antecubital vein, at a rate of 2.5 mL/s by an MRI-compatible automated injector pump, followed by an equal volume of normal saline solution

### CT scanning techniques

The imaging protocol is shown in Table 3. CT scanning includes the venous phase at 50 s after the injection of the contrast medium. CT scanning parameters are as follows: Voltage = 120 KV, tube current = 300 mAs.

**Table 3:** Key technical parameters of esophageal CT

Section thickness (mm)	Section spacing (mm)	Voltage (kV)	Tube current (mAs)	Detector	Pitch	FOV (mm)	Delay time	Dose of contrast medium (mL/kg)	Rate of contrast medium injection (mL/s)
5	5	120	300	128 × 1.0	0.993	396 × 396	50 s	1.5–2.0	2.0–2.5

### Malignant solid masses

Malignant esophageal solid masses include esophageal cancer, which can cause an esophageal-airway fistula that also seems like a mass, esophageal neuroendocrine carcinoma, esophageal carcinosarcoma, esophageal lymphoma, and esophageal malignant melanoma. With the development of MR techniques, the different layers of the esophageal wall can be displayed clearly, and from which layers masses originate can be observed. MRI is superior to CT in demonstrating tiny necrosis, homogeneous intensity, especially on both T2-weighted images and contrast-enhanced T1-weighted images for lymphoma, and hyperintense T1 with hypointense T2-weighted images for typical esophageal malignant melanoma. It is necessary to know what MRI can provide for esophageal masses.

### Esophageal cancer

Esophageal carcinoma stands as one of the most fatal malignant tumors globally, ranking as the sixth most predominant factor contributing to mortality. CT traditionally serves as a means to assess potential resectability, yet has limitations when it comes to delineating the distinct layers of the esophageal wall. Recent investigations have delved into the employment of MRI for T staging in esophageal cancer. Compared to CT, MRI showed significantly higher accuracy (96% vs 82%, *p* = 0.0038, for MRI vs CT), and contrast-enhanced radial 3D-GRE images and T2W TSE have proven instrumental in effectively illustrating the different layers comprising the esophageal wall [3, 5, 7–9]. Normal mucosa shows hyperintensity on the T2-weighted image, intensely homogeneous enhancement on the arterial phase, and no restricted diffusion. Normal muscularis propria appears hypointense on the T2-weighted image with slightly homogeneous enhancement on venous and delayed phases and no restricted diffusion.

**Fig. 1:**
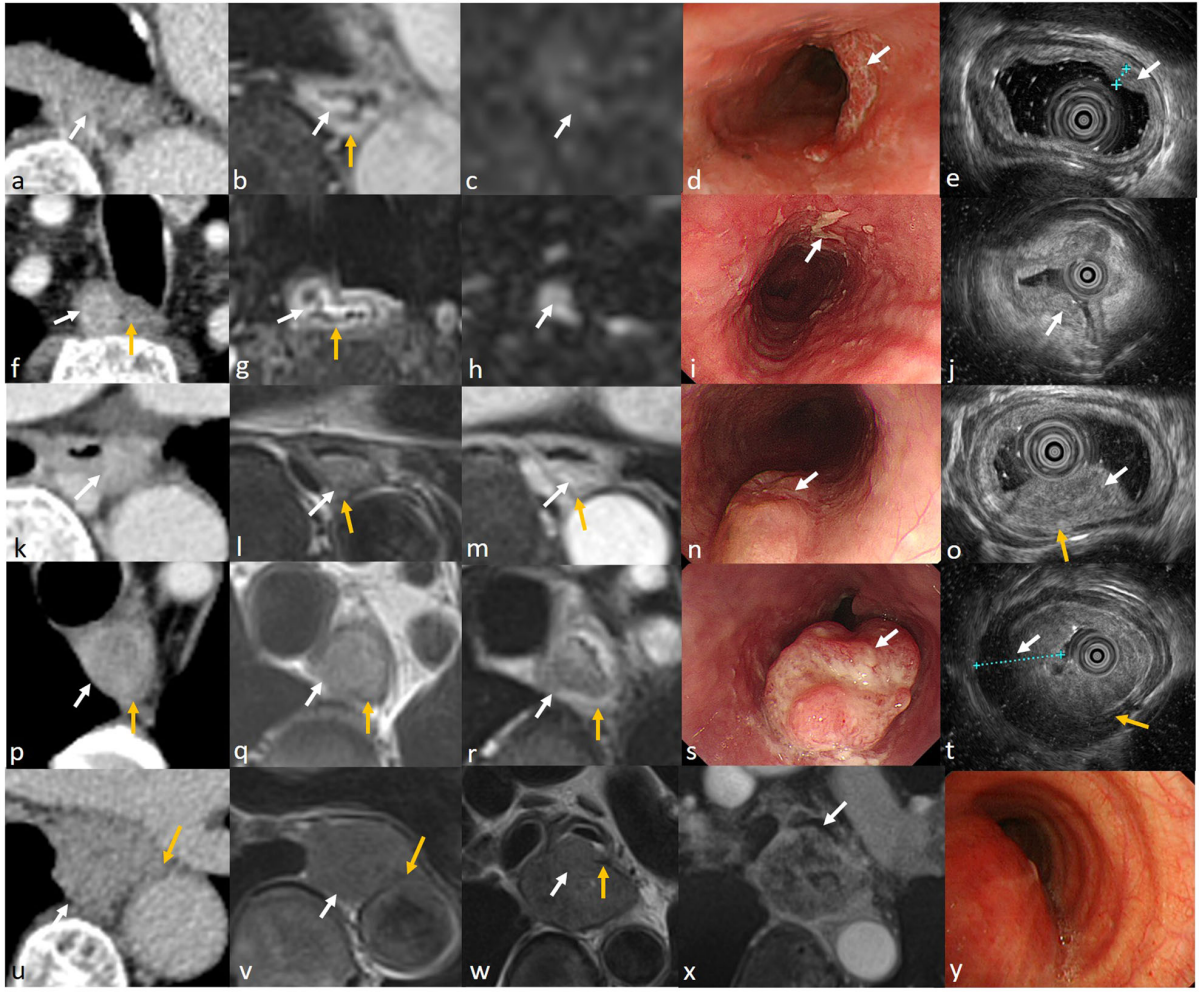
Stage T1-4 esophageal cancer on MR imaging. Stage T1a (**a**–**e**), it is difficult to detect the lesion on contrast-enhanced CT image (**a**). The lesion shows an intensely enhancing homogenous pedunculated mass (white arrow), with a degree of enhancement similar to that of the mucosa (yellow arrow) after contrast injection on T1-weighted image (**b**) and no restricted diffusion (**c**). Stage T1b (**f**–**j**), the lesion shows heterogeneously enhancing mass (white arrow) on contrast-enhanced CT image (**f**), similar to normal mucosa (yellow arrow), tumor blurs intensely enhanced mucosa after contrast injection on T1-weighted image (**g**) and has restricted diffusion (**h**). Stage T2 (**k**–**o**), it is difficult to detect the lesion on contrast-enhanced CT image (**k**). The tumor (white arrow) invades muscularis propria (yellow arrow), which is blurred on the T2-weighted image (**l**) and on the contrast-enhanced T1-weighted image (**m**). Stage T3 (**p**–**t**), the lesion (white arrow) appears to invade muscularis propria (yellow arrow) on contrast-enhanced CT image (**p**), however, the tumor involves muscularis propria and invades adjacent fat and fibrosis tissue. The muscularis propria is interrupted on the T2-weighted image (**q**) and on the contrast-enhanced T1-weighted image (**r**). Stage T4 (**u**–**x**) in two cases, the tumor (white arrow) appears to invade the aorta (yellow arrow) on contrast-enhanced CT image (**u**). The tumor invades the aorta on the T2-weighted image (**v**). The hypointense adventitia of the left principal bronchus (yellow arrow) is interrupted on the T2-weighted image (**w**) and on the contrast-enhanced T1-weighted image (**x**). The lesion is shown in the oesophagoscope (**d**, **I**, **n**, and **s**) and endoscopic ultrasonography (**e**, **j**, **o**, and **d**). The bronchoscope(y) shows the lesion invades the outer membrane of the left principal bronchus, while the inner membrane is intact

T stage is typically performed on MRI using the revised Vienna classification of gastrointestinal epithelial neoplasia and the 8th edition AJCC/UICC staging of cancers of the esophagus [7, 10, 11]. MRI criteria for T staging of esophageal cancer are shown in Table 4 and MR features of different T staging are displayed in Fig. 1.

**Table 4:** MRI criteria for T staging of esophageal cancer

Stage MRI criteria	Shape	T2-weighted image	Contrast-enhanced T1-weighted image	DWI
MHN	Thickening mucosa or focal mass with pedunculated “heart-shaped” appearance	Iso intensity, compared with normal mucosa	Intensely homogeneous enhancement and intact mucosa	No restricted diffusion
T1b	Mass	Iso intensity, compared with normal mucosa	Sightly heterogeneous hypoenhancement, which is lower than normal mucosa, and mucosa is blurred	Restricted diffusion
T2	Mass	Slight hyperintensity, compared with muscularis propria, mass invades muscularis propria, but not interrupted	Hypoenhanced mass invades mucosa and blurred muscularis propria	Restricted diffusion
T3	Mass	Lower-signal muscularis propria is interrupted, and adjacent fat and fibrosis is invaded	Hypoenhanced mass breaks mucosa and muscularis propria, and invades adjacent fat and fibrosis	Restricted diffusion
T4	Mass	Lower-signal muscularis propria is interrupted, and the adjacent organ is invaded	Hypoenhanced mass invades adjacent organs	Restricted diffusion

*MHN* mucosal high-grade neoplasia

T1: the tumor is located in the mucosa and the mucous layer remains ring-like intact;

Mucosal high-grade neoplasia: including high-grade adenoma/dysplasia, noninvasive carcinoma, suspicious for invasive carcinoma, and intramucosal carcinoma according to the revised Vienna classification of gastrointestinal epithelial neoplasia [7, 10], meanwhile included Tis and T1a according to the 8th edition AJCC/UICC staging of cancers of the esophagus [11].

T1b, an early invasive cancer, which is submucosal invasion by carcinoma [7].

T2: tumor invades muscularis propria, but without breaking through muscularis propria;

T3: the tumor breaks through muscularis propria and invades adjacent fat and fibrosis.

T4: tumor invades adjacent structures [3, 8].

### Esophageal fistula

**Fig. 2:**
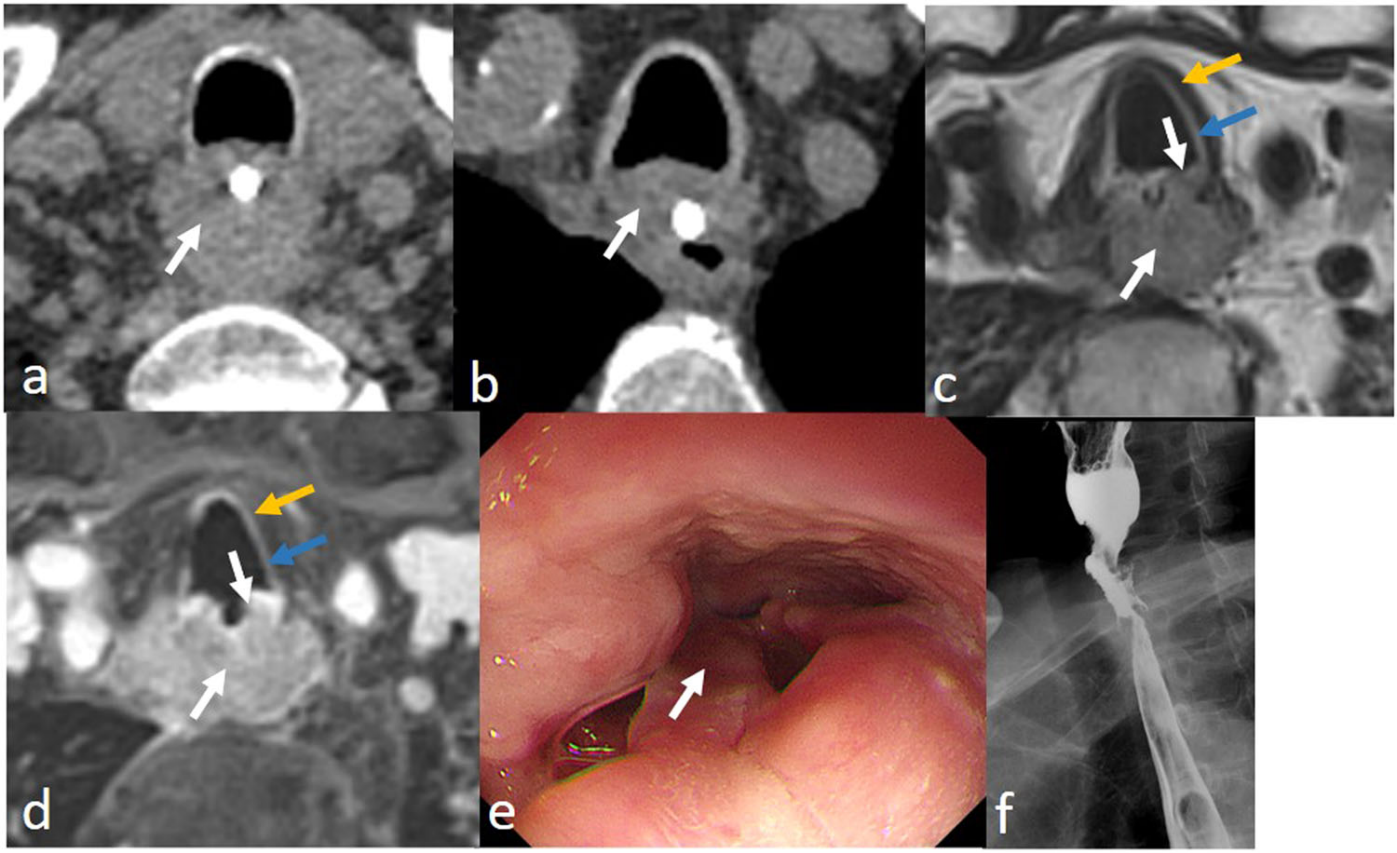
Esophageal fistula in a 62-year-old man. A tumor (white arrow) is poorly defined on CT and tracheal involvement is not confirmed (**a**, **b**). MRI clearly delineates that the tumor invades the trachea. The hypointense adventitia (yellow arrow) and hyperintense intima (blue arrow) of the trachea are interrupted in the T2-weighted image (**c**) and in the contrast-enhanced T1-weighted image (**d**). The tumor obstructs the entire esophagus, making it impossible for the oesophagoscope or endoscopic ultrasonography to pass through (**e**). Barium esophagogram shows the esophageal fistula (orange arrow)

Esophageal-airway fistula is a complication of esophageal cancer or secondary to esophageal trauma, infections, or radiochemotherapy. More than half of such fistulae involve the trachea, and a connection with the main or lower lobe bronchus, pleura, pericardium, or mediastinal fat may be formed. CT is necessary to localize the fistula and can also be used to detect pleuro-pulmonary or mediastinal inflammatory reactions to it [12, 13]. However, for tiny mediastinal fistulae or if the fistula involves only the adventitia of the trachea, MRI is superior to CT in displaying the detailed features (Fig. 2). MRI can demonstrate two layers of the tracheal wall—the intima, which consists of mucous membrane and the submucosa layer, and the adventitia, consisting of hyaline cartilage ring, trachealis muscle, and connective tissue. These layers are difficult to display on CT.

### Esophageal neuroendocrine carcinoma (ENEC)

**Fig. 3:**
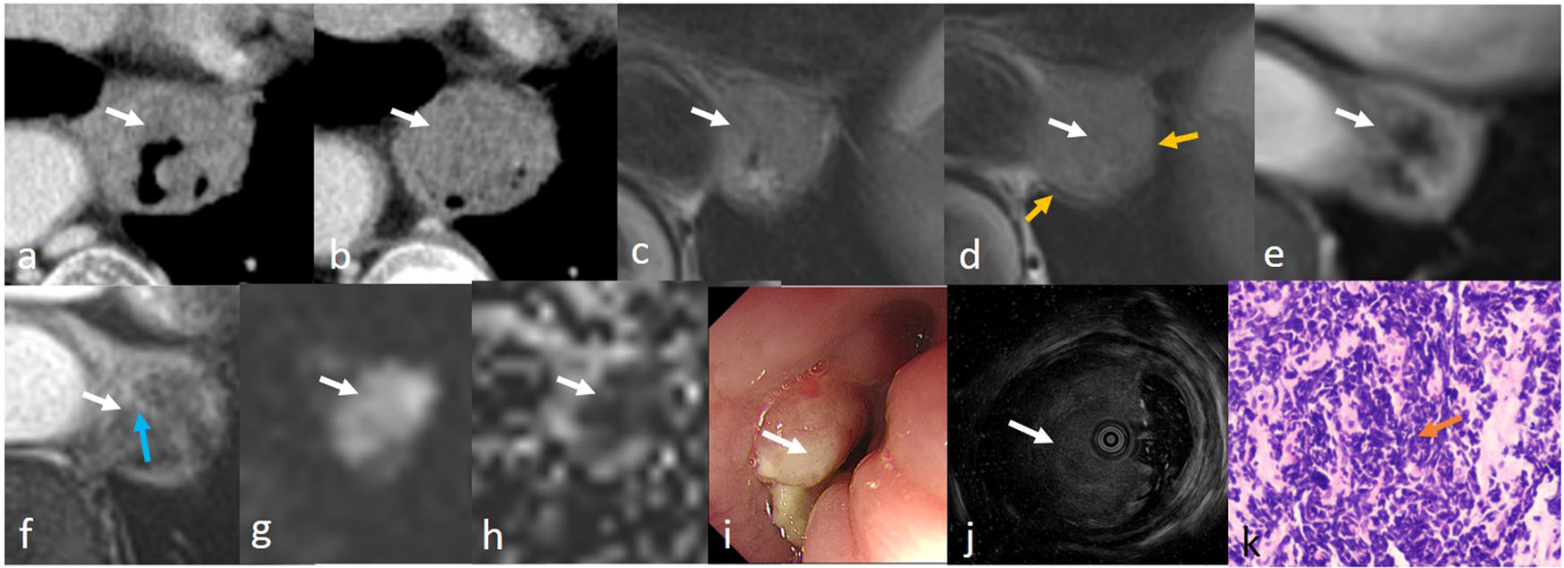
ENEC images in a 67-year-old woman. Lesion (white arrow) shows heterogeneous slightly enhancing mass after contrast injection on CT image (**a**, **b**). The lesion is relatively homogeneous slightly hyperintensity, and hypointense muscularis propria (yellow arrow) is interrupted on T2-weighted images (**c**, **d**). The lesion has slightly heterogeneous enhancement with intensely enhancing stalk (blue arrow) on contrast-enhanced T1-weighted images (**e**, **f**), restricted diffusion (**g**), and low ADC value (mean: 1.318 × 10^−3^ mm^2^/s) (**h**). The lesion is shown in oesophagoscope (**i**) and endoscopic ultrasonography (**j**). H&E-stained section at × 200 microscopies confirmed the presence of esophageal neuroendocrine carcinoma (ENEC) (orange arrow) (**k**) with CD56 (neuronal cell marker) ( + ), CgA (neuroendocrine marker) ( + ), SyN (synaptophysin, synapses marker) ( + )

**Fig. 4:**
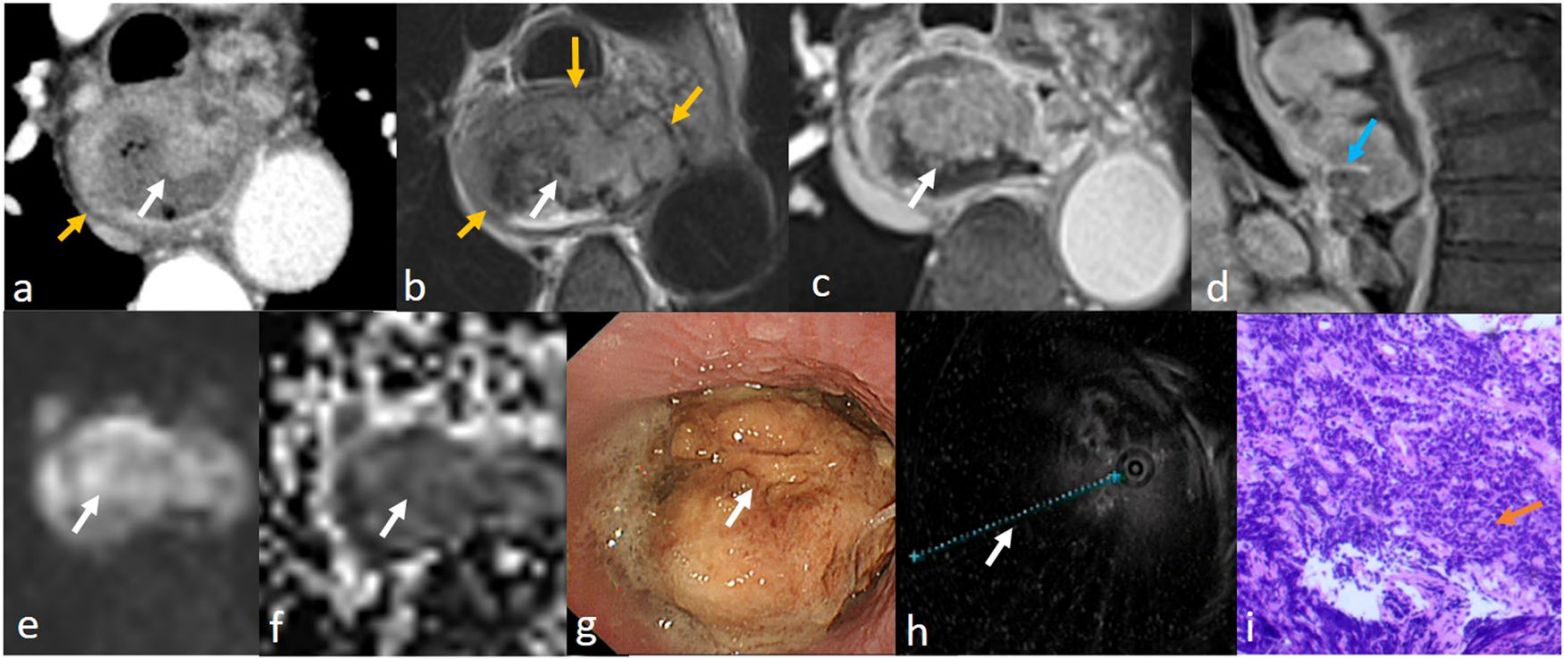
ENEC images in a 63-year-old man. Lesion (white arrow) shows large heterogeneous enhancing mass after contrast injection on CT image (**a**). The mass is heterogeneous and slightly hyperintense on the T2-weighted image. The hypointense muscularis propria (yellow arrow) is interrupted (**b**). The mass is heterogeneous and moderately enhancing with intensely enhancing stalk (blue arrow) (**c**, **d**) on post-contrast T1-weighted image, restricted diffusion (**e**), and low ADC value (mean: 0.815 × 10^−3^ mm^2^/s) (**f**). The lesion is shown in oesophagoscope (**g**) and endoscopic ultrasonography (**h**). H&E-stained section at × 100 microscopy confirmed the presence of ENEC (orange arrow) by biopsy (**i**) with CD56 (neuronal cell marker) ( + ), CgA (neuroendocrine marker) ( + ), SyN (synaptophysin, synapses marker) ( + ). CD56 can be used as a biomarker to detect neuroendocrine carcinoma. CgA is widespread in neuroendocrine cells and is found in almost all types of neuroendocrine tumors. SyN can be used as a marker for neuroendocrine cells. This patient received nCT and TRG 0 after surgery

ENEC is a rare disease with aggressive progression and extremely unfavorable prognosis, accounting for 0.05–3.1% of all esophageal cancers [14]. For neuroendocrine carcinoma, the esophagus is the most common location in the digestive system [15]. Most ENECs show well-defined tumor margins, central necrosis, heterogeneous enhancement, and moderate to markedly intense enhancement. However, these are non-specific imaging manifestations [16]. MRI could provide more information than CT, including accurate T staging and more detailed tumor features (Figs. 3, 4).

### Esophageal carcinosarcoma

**Fig. 5:**
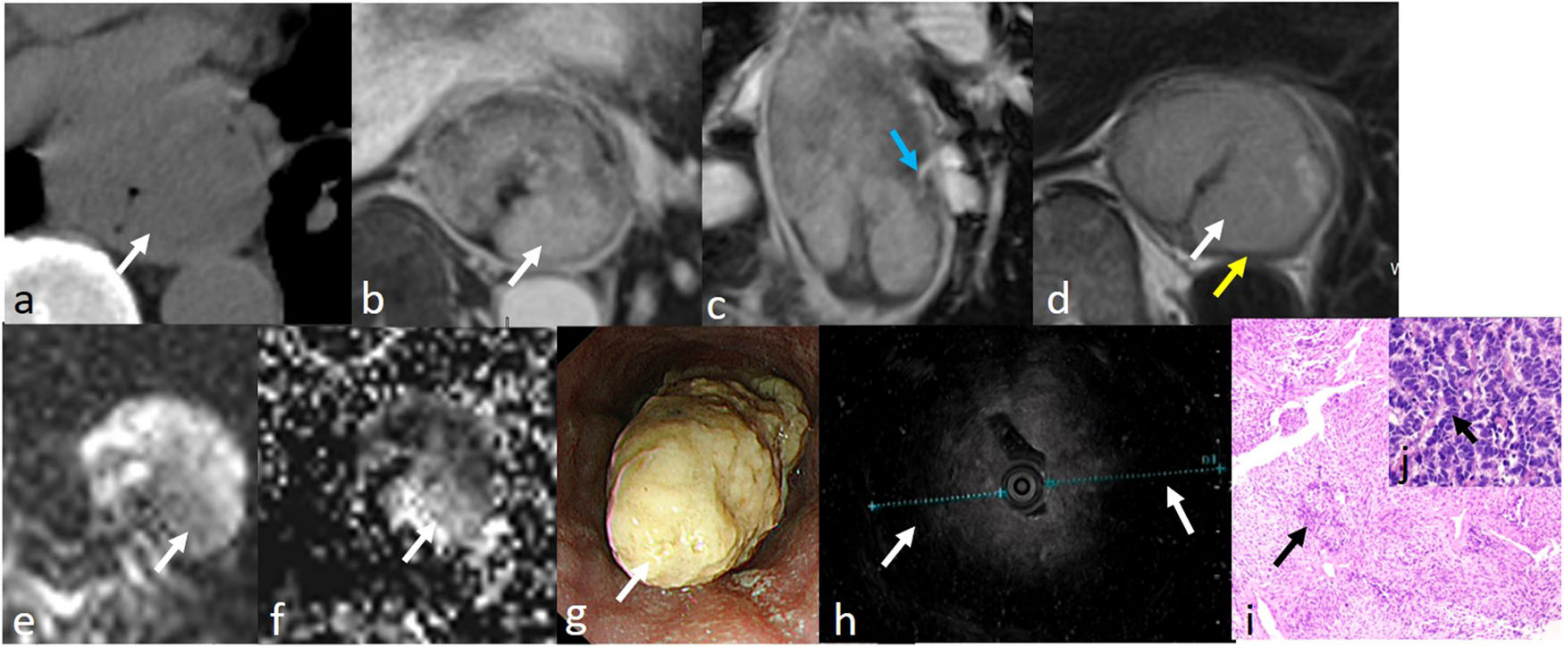
Mixed carcinosarcoma and poorly differentiated neuroendocrine carcinoma images in a 64-year-old man. The lesion is large on CT image (**a**), and is slightly enhancing (white arrow) with enhancing stalk (blue arrow) on contrast-enhanced T1-weighted images (**b**, **c**). the mass is slightly hyperintense compared with muscularis propria (yellow arrow) on the T2-weighted image and has restricted diffusion (**e**) and low ADC value (mean: 0.523 × 10^−3^ mm^2^/s) (**f**). The lesion is shown in oesophagoscope (**g**) and endoscopic ultrasonography (**h**), and almost obstructs the esophagus completely. H&E-stained section at × 200 microscopy confirms the presence of esophageal cancerous sarcoma (black arrow) (**i**) with Vimentin (mesenchymal cell marker) ( + ). H&E-stained section at × 200 microscopy confirms the presence of ENEC (gray arrow) with CD56 ( + ), CgA ( + ), SyN ( + ) (**h**)

**Fig. 6:**
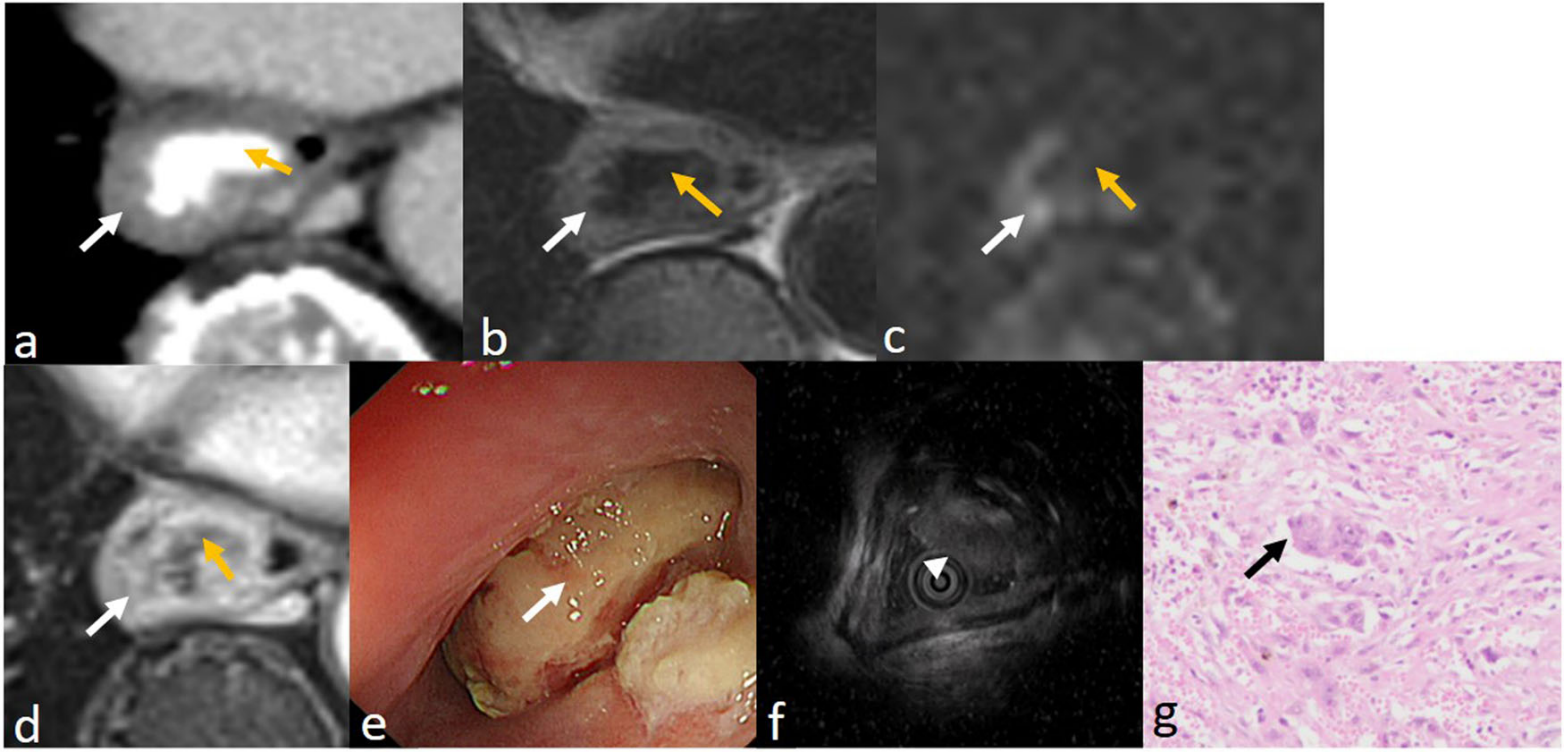
Esophageal carcinosarcoma in a 68-year-old woman. The lesion shows moderate inhomogeneous enhancing mass (white arrow) with coarse calcification (yellow arrow) on contrast-enhanced CT image (**a**). The mass is slightly hyperintense than muscularis propria on the T2-weighted image with a small focus of lower-signal intensity (yellow arrow) (**b**). The solid part shows restricted diffusion (white arrow) with the focus of calcification showing no diffusion restriction and no restricted diffusion (yellow arrow) (**c**). The mass shows heterogeneous enhancement on post post-contrast T1-weighted image, except for the focus of calcification which is not enhancing (**d**). The lesion is shown in oesophagoscope (**e**) and endoscopic ultrasonography (**f**). H&E-stained section at × 200 microscopy confirms the presence of esophageal cancerous sarcoma (green arrow) with Vimentin ( + ) (**g**)

Esophageal carcinosarcoma is a relatively rare malignant tumor consisting of carcinomatous and sarcomatous components, and it accounts for 0.5–2.8% of all esophageal malignancies [17]. The sarcomatous component forms a large intraluminal pedunculated mass that consists of pleomorphic atypical histiocyte-like cells, osseous, cartilaginous, and/or skeletal-muscular components are occasionally observed, which indicates overt mesenchymal differentiation [18]. Esophageal carcinosarcoma usually presents as a large intraluminal pedunculated, polypoid mass [19]. The mass often shows an ill-defined, intraluminal, solid mass with occasional hyperdense osseous on unenhanced chest CT images. moderate inhomogeneous enhancement on contrast-enhanced CT images. Most tumors present as a polypoid mass with a pedicle. Carcinosarcomas have a lower tendency to invade nearby organs, even late in their course [20]. Other histological components, such as neuroendocrine carcinoma, squamous cell carcinoma, and sarcoma, are rarely observed. Figure 5 shows a case with mixed carcinosarcoma and poorly differentiated neuroendocrine carcinoma, and Fig. 6 shows a case with osseous components. Although both tumors are large, they only invade the layer of muscularis propria without invading nearby organs.

### Esophageal lymphoma

Primary esophageal lymphoma is rare, and fewer than 25 cases have been reported in the literature. Theoretically, any histologic variety of lymphoma may affect the esophagus, however, the esophagus is the least commonly involved organ in the alimentary track [21]. CT may demonstrate a homogeneously enhancing mass with sharply delineated or irregular borders, pronounced polypoid wall thickening in any part of the esophagus, with or without associated lymphadenopathy. However, there is no specific CT finding for esophageal lymphoma [22]. MRI has the ability to display the homogeneous intensity, especially on both T2-weighted images and contrast-enhanced T1-weighted images, and the relations with different layers of the esophageal wall.

### Esophageal malignant melanoma

Primary esophageal malignant melanoma is a highly aggressive, but very rare tumor that accounts for 0.1–0.4% of all esophageal malignancies [23]. To date, less than 300 primary esophageal melanomas have been reported in the literature [24]. Esophageal malignant melanoma presents exophytic tumor or pedunculated tumor and flat pigmented area. Tumor has uniform CT density, and varying degrees of enhancement [25, 26], but CT is not helpful in identifying properties of esophageal melanomas. The appearance of melanoma on MRI has been well described, and many theories have been put forth to explain the reason for the T1 and T2 shortening [26]. Typical esophageal malignant melanoma shows hyperintensity on T1-weighted images and hypointensity on T2-weighted images (Fig. S1).

## Benign tumor and tumor-like lesion

### Esophageal leiomyoma

Esophageal benign neoplasms are rare, and esophageal leiomyoma accounts for 60–70% of all benign neoplasms, while leiomyoma is rare in the remaining gastrointestinal tract [27*–*29]. Esophageal leiomyoma presents more often in male patients (2:1) at a median age of 30–35 years. It is usually intramural in location, within the esophageal wall in 97% of all reported cases, whereas true polypoid tumor was found in only 1%. The remaining 2% demonstrated extra-esophageal extension, as mediastinal outgrowth [28]. Leiomyoma may appear as a well-circumscribed sessile solid mass, occasionally pedunculated, polypoidal, or exophytic intraluminal solid masses, sometimes with secondary ulceration. The absence of the typical circumferential growth pattern or infiltration of the esophageal wall enables differentiation from esophageal cancer [22].

Leiomyomas generally occur as intramural eccentric lesions [30]. Usually, leiomyomas are between 2 and 8 cm in diameter. CT findings include a smooth or lobulated tumor margin, with either iso or homogeneously low attenuation on contrast-enhanced CT images. Leiomyoma has a similar intensity to muscularis propria, with iso intensity on the T2-weighted image, no diffusion restriction, and slightly homogenous enhancement on the contrast-enhanced T1-weighted image (Fig. S2). Lack of restricted diffusion may help differentiate esophageal leiomyoma from gastrointestinal stromal tumors (GISTs) and esophageal cancer [31].

### Esophageal gastrointestinal stromal tumors

GISTs are the most common mesenchymal tumors in the gastrointestinal tract, with 1–3% GISTs occurring in the esophagus. They have a known malignant potential, like that of GISTs elsewhere in the gastrointestinal tract, and they are similar to esophageal leiomyomas on imaging. Esophageal GISTs are typically larger than esophageal leiomyomas, often measuring greater than 10 cm. These tumors are more consistent in the distal esophagus, more heterogeneous in density or intensity, and have greater enhancement than leiomyoma. Calcifications were rare for both lesions, occurring in two of ten esophageal leiomyoma patients and in one of eight esophageal GIST patients [32]. Calcifications were described as diffuse popcorn-type appearance in the two esophageal leiomyomas, but a focal eccentric coarse appearance in the one esophageal GIST [33]. Esophageal GISTs appear as bulky enhancing heterogeneous FDG-avid masses [33] with diffusion restriction in MRI (Fig. S3). These imaging features may facilitate the differentiation of esophageal GISTs and leiomyomas.

### Esophageal schwannoma

Schwannomas account for 2–6% of gastrointestinal mesenchymal tumors and usually originate in the stomach or intestine. Esophageal schwannoma is rare [34]. Although esophageal schwannoma has both benign and malignant features; “schwannoma” usually refers to benign tumors [35]. The rate of misdiagnosis and mistreatment is high because of the lack of awareness of the esophageal schwannoma and the need for pathological diagnosis [36]. The esophageal wall is soft, and the mucosa remains intact. The reported tumor diameter is 35–110 mm and an average of 64.8 ± 24.4 mm, in 18 cases [35]. Tumors usually display low or equal attenuation to the esophageal wall, uniform texture, occasional presence of calcified areas, and different degrees of enhancement on contrast-enhanced CT images (Fig. S4). Esophageal malignant schwannomas display more heterogeneity on unenhanced and contrast-enhanced CT images and might have infiltrates with blurred boundaries [37]. The tumors show equal or slightly higher intensities than muscles on T1-weighted images [38]. Esophageal schwannoma has high-intensity edges with low-intensity centers, doesn’t invade the surrounding tissue on T2-weighted images [39], and may be accompanied by peritumoral lymphoid cuff, which is significantly associated with regional lymph node enlargement [40].

### Esophageal lipoma

Esophageal lipomas account for less than 1% of all benign esophageal neoplasms. They can present as an intramural submucosal mass, or as an intraluminal mass with a long and narrow pedicle covered by intact mucosa [41]. Esophageal lipomas consistently share the presence of variable amounts of mature adipocytes and fibrovascular septa, so these neoplasms are also described and reported as fibrovascular polyp, fibrolipoma, or angiolipoma [42]. Both CT and MRI can effectively demonstrate the fatty composition of these neoplasms, which manifests as low attenuation in CT images and high signal intensity in MR images, as well as low or suppressed signal intensity in fat saturation images. (Fig. S5). When the signals or density become uneven, the morphology becomes irregular, and the enhancement is heterogeneous, it indicates the malignant transformation of lipoma.

### Esophageal hemangioma

Esophageal hemangioma is a rare entity, with few cases reported [43], and the most common location is reported to be the cervical esophagus [44]. Esophageal hemangioma can mimic a large esophageal polyp [43]. Contrast-enhanced CT reveals a poorly defined enhancing mass but is well separated from adjacent tissue. Contrast-enhanced MRI usually demonstrates a submucosal, homogeneous, strong, or gradually enhancing, mass with iso intensity to mucosa on both T2-weighted images and contrast-enhanced T1-weighted images (Fig. S6). The tumor can also show poor enhancement or slow gradual enhancement, and some nodular calcifications and peripheral puddling of contrast medium [45, 46].

### Fungal esophagitis

The most common cause of infectious esophagitis is candida, with an incidence of up to 88% [47]. Fungi proliferate in esophageal mucosa and form adhesive plaques [48]. Double-contrast esophagography can present the characteristic manifestations of esophageal stenosis, as “foamy appearance” and “feather appearance” [47, 49], with a sensitivity of up to 90% [48]. Fungal esophagitis manifests with expansive wall thickening exceeding 5 mm, showcasing a picturesque configuration of the wall due to the presence of enhanced mucosa and submucosa with decreased density. This condition encompasses esophageal ulceration and the formation of fistulas. Furthermore, intramural pseudodiverticulosis may also be observed. In the context of an appropriate clinical presentation, the identification of an elongated section of concentric and circumferential wall thickening indicates the likelihood of esophagitis. [50]. The radiographic manifestations of a fungal-infected esophageal cyst case from our center are as follows: CT images reveal a homogenous non-enhancing mass, while MR images show a submucosal cyst in the esophagus with moderate heterogeneous hyperintensity which is slightly higher than mucosa on T2-weighted image, no diffusion restriction, and homogenous unenhanced mass after contrast injection on MR image (Fig. S7).

## Conclusions

MRI plays a crucial role in a detailed assessment of the esophageal wall layers, offering new opportunities for early detection and accurate characterization of esophageal lesions, whether benign or malignant. The future advancements emerging in MR imaging techniques are expected to further enhance the diagnosis and management of esophageal diseases, improving the sensitivity and specificity in identifying esophageal masses, and guiding more precise treatment strategies. Therefore, the ongoing development of MR imaging and the identification of imaging characteristics of different esophageal diseases will contribute to the flourishing of MRI technology in the field of esophageal diseases.

### Supplementary information

ELECTRONIC SUPPLEMENTARY MATERIAL

## Data Availability

The datasets used or analyzed during the current study are available from the corresponding author on reasonable request.

## Abbreviations

DWI: Diffusion-weighted imaging
ENEC: Esophageal neuroendocrine carcinoma
GISTs: Gastrointestinal stromal tumors
T2W TSE: T2 weighted turbo spin-echo
