# Supplementary material for: How to update esophageal masses imaging using literature review (MRI and CT features)
Source: Insights Imaging. 2024 Jul 6;15:169. doi: 10.1186/s13244-024-01754-0 (PMC11227487; doi:10.1186/s13244-024-01754-0)

How to update esophageal masses imaging using literature review (MRI and CT features)

ELECTRONIC SUPPLEMENTARY MATERIAL

Insights Imaging (2024) Qu JR, Wang ZG, Zhang HK, et al.

Figure S1. Esophageal malignant melanoma images in an 81-year-old woman. Lesion shows homogeneous isodensity on contrast-enhanced CT image (a). The lesion is heterogenous hyperintense on pre-contrast T1-weighted image (b), no definite enhancing mass (white arrow) (c) with intensely enhancing stalk (yellow arrow) on contrast-enhanced T1-weighted image (d), hypo- and slightly hyper-intensity on T2-weighted image (e), restricted diffusion and lower ADC value (mean: $1.595 \times 10^{-3} mm^2/s$) (f, g). The lesion is showed in oesophagoscope (h) and endoscopic ultrasonography (i), with features of melanin or recent bleeding. H&E-stained section at ×100 microscopy confirms the presence of esophageal malignant melanoma with diffuse proliferation of round cells, non-cohesive, with intracytoplasmic melanic pigment (green arrow) (j).

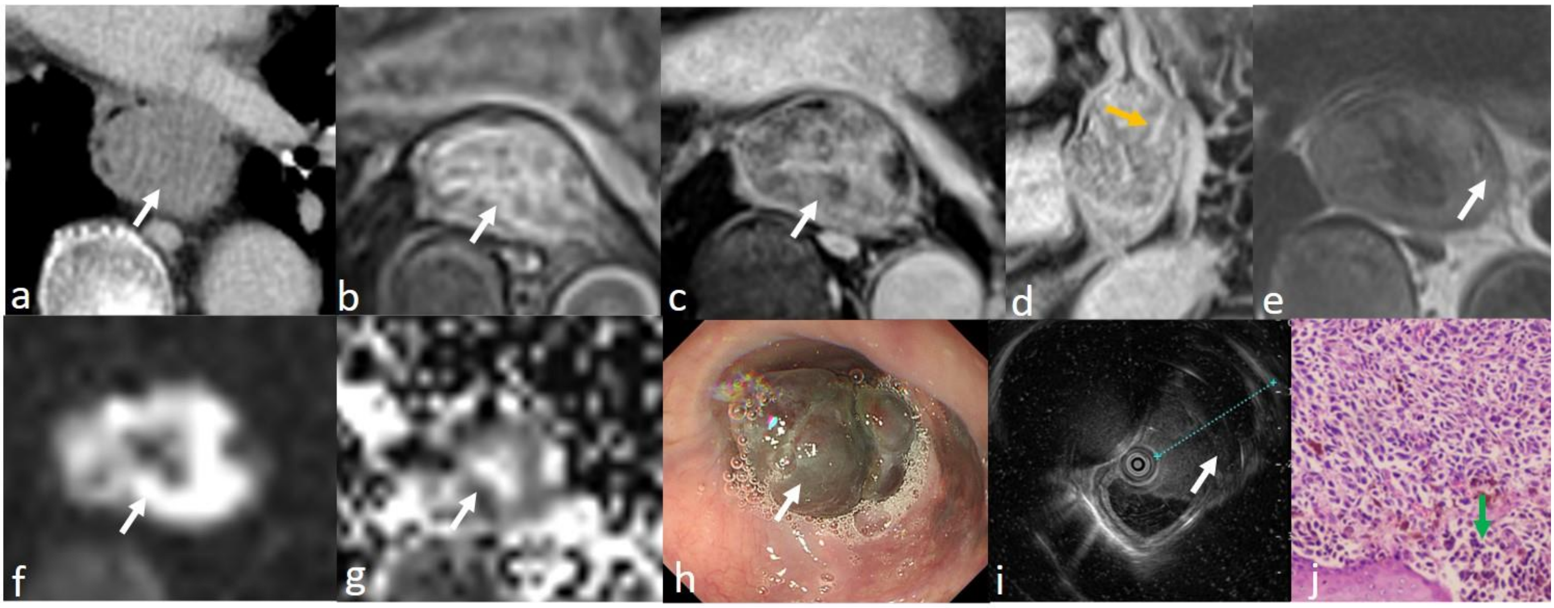

Figure S2. Esophageal leiomyoma in a 45-year-old woman. There is iso attenuating mass (white arrow) on contrast enhanced CT image (a). The mass is, isointense to muscularis propria on T2-weighted image with similar intensity to muscularis propria (yellow arrow) (b), slightly enhancing on contrast enhanced T1-weighted image (c), no diffusion restriction (d), and iso ADC value to muscularis propria (mean: $1.035 \times 10^{-3} mm^2/s$) (e). Oesophagoscope (f) and endoscopic ultrasonography (g) shows this submucous protrusion lesion. H&E-stained section at × 200 microscopy confirms the presence of leiomyoma with rod cell nucleus (blue arrow) (h).

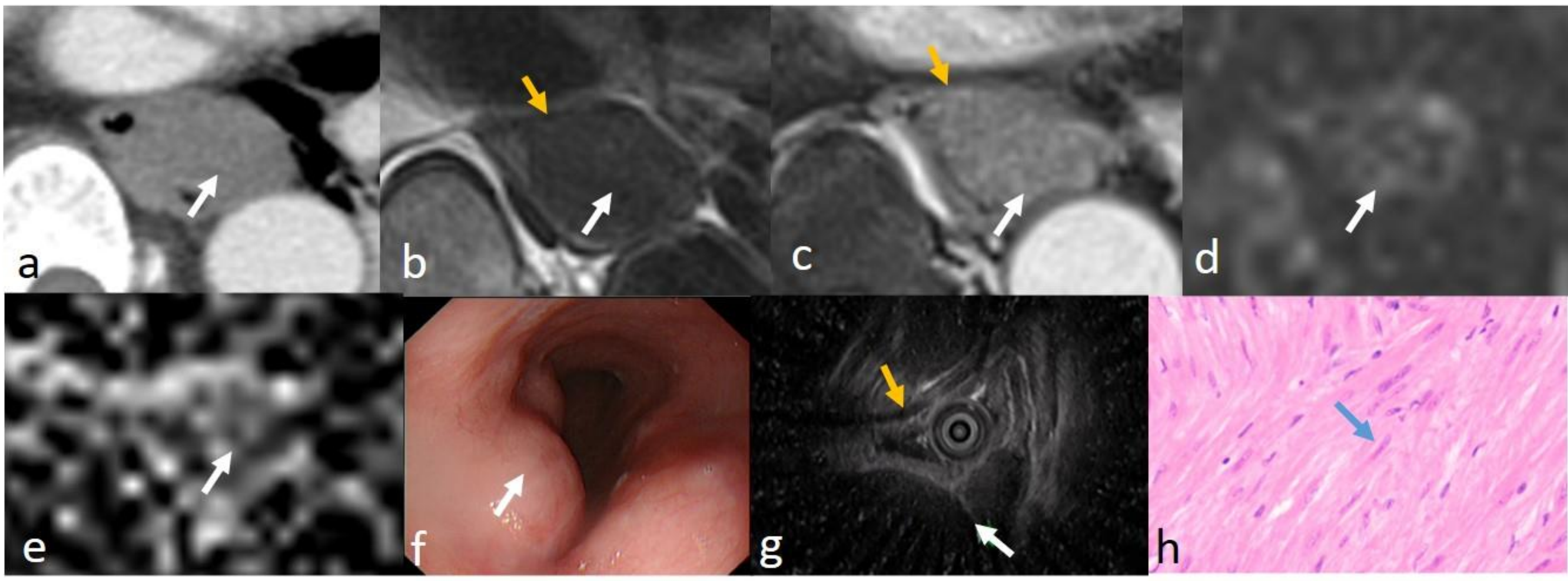

Figure S3. Esophageal distal GISTs images in a 66-year-old man. Lesion shows heterogeneously enhancing mass (white arrow) with unenhanced necrosis (blue arrow) on contrast-enhanced CT (a). The mass is, slightly hyperintense, compared to muscularis propria (yellow arrow), with areas of necrosis on T2-weighted image (b). there is, heterogeneous enhancement with areas of necrosis on contrast enhanced T1-weighted image (c). Solid part (red arrow) shows diffusion restriction (d) and low ADC value (mean: $1.441 \times 10^{-3} mm^2/s$) (e), while necrotic part shows no diffusion restriction and high ADC value. Oesophagoscope (f) and endoscopic ultrasonography (g) shows this submucous protrusion lesion, which is very similar to esophageal leiomyoma. H&E-stained section at × 100 microscopy confirms the presence of GIST with long shuttle tumor cells (blue arrow), and CD34 (hematopoietic stem cell & endothelial marker) (+), cd117 (GIST marker) (+), Dog-1(GIST marker) (+) (h).

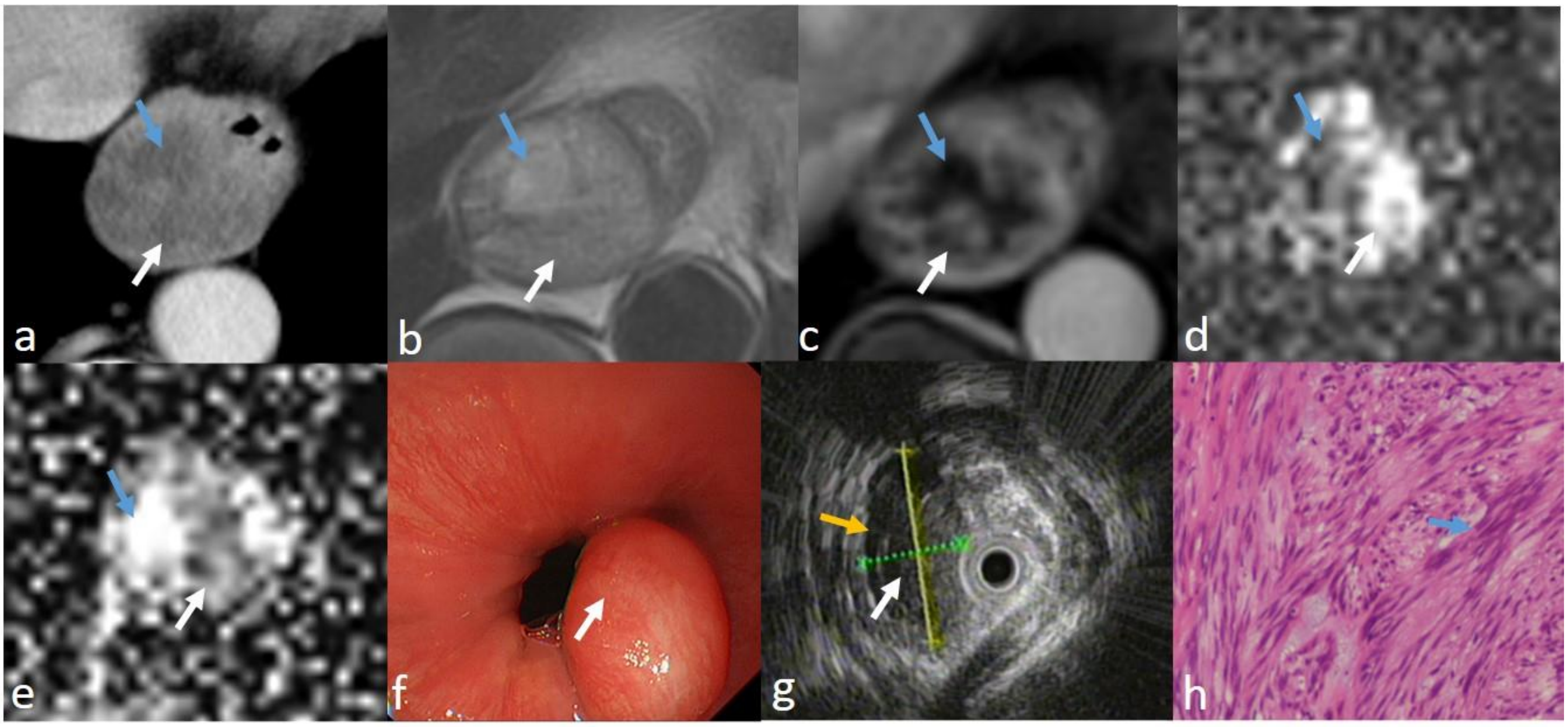

Figure S4. Esophageal schwannoma in a 71-year-old man. Axial CT images (a, b) and multiplanar reformation CT images (c, d) show a large lesion of the esophagus with heterogenous enhancement (white arrow), and a second sessile mass with thinning enhanced mucosa (yellow arrow) on contrast-enhanced CT images. Axial CT image show peritumoral lymph node enlargement (green arrow, e). Oesophagoscope (f) and endoscopic ultrasonography (g) shows the lesion almost blocks the entire esophagus. H&E-stained section at ×200 microscopy confirms the presence of schwannoma with shuttle tumor cells, and S-100 (schwannoma marker) (+), SOX-10 (schwannoma marker) (+) (h).

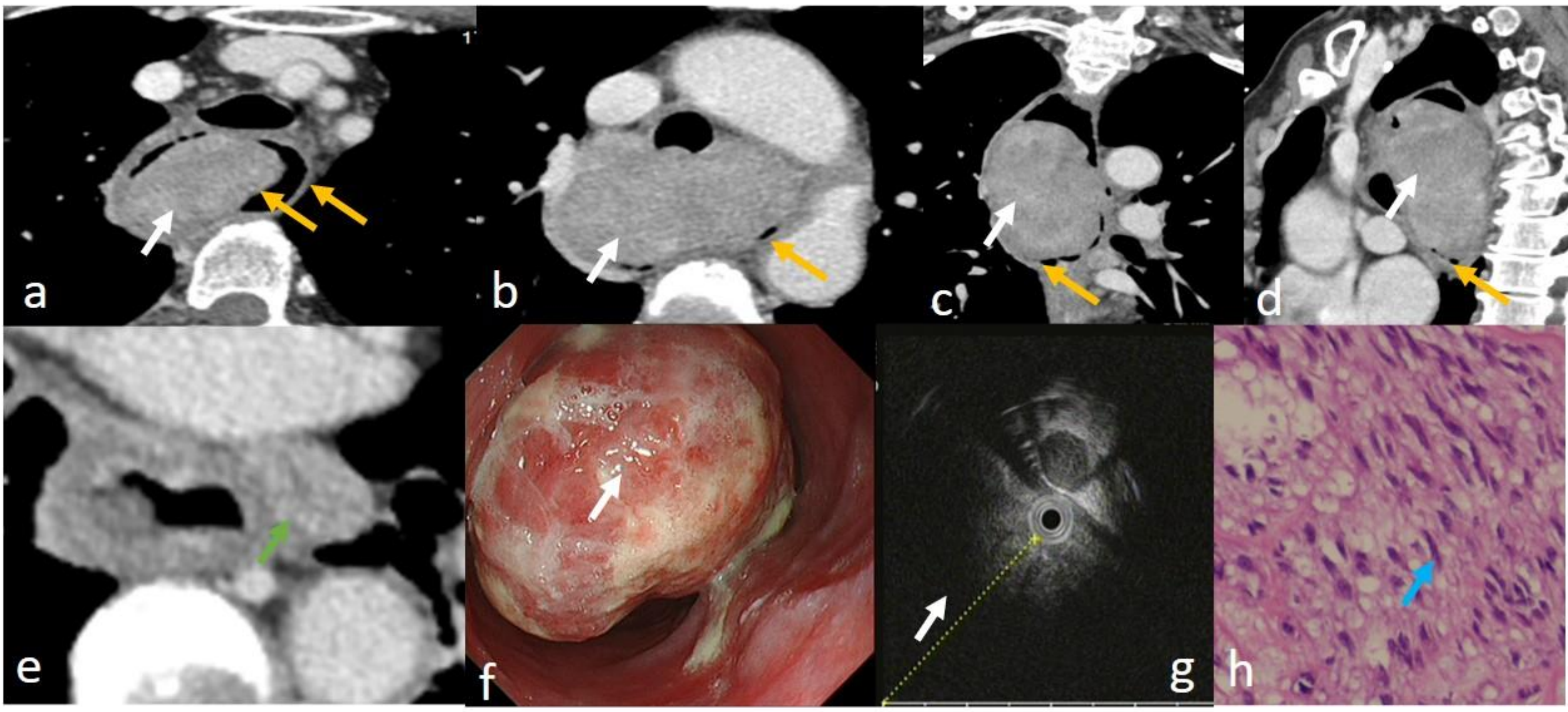

Figure S5. Esophageal lipoma images in a 58-year-old woman. Lesion shows low attenuation and unenhanced mass (yellow arrow) after contrast injection on CT image (a), submucosal high intensity mass (yellow arrow) on both T1- and T2-weighted images (b, c), unenhanced low intensity mass after contrast injection on fat saturation T1-weighted image. Oesophagoscope (f) and endoscopic ultrasonography (g) shows this little submucous protrusion lesion, endoscopic resection is performed subsequently to show adipose tissue (g).

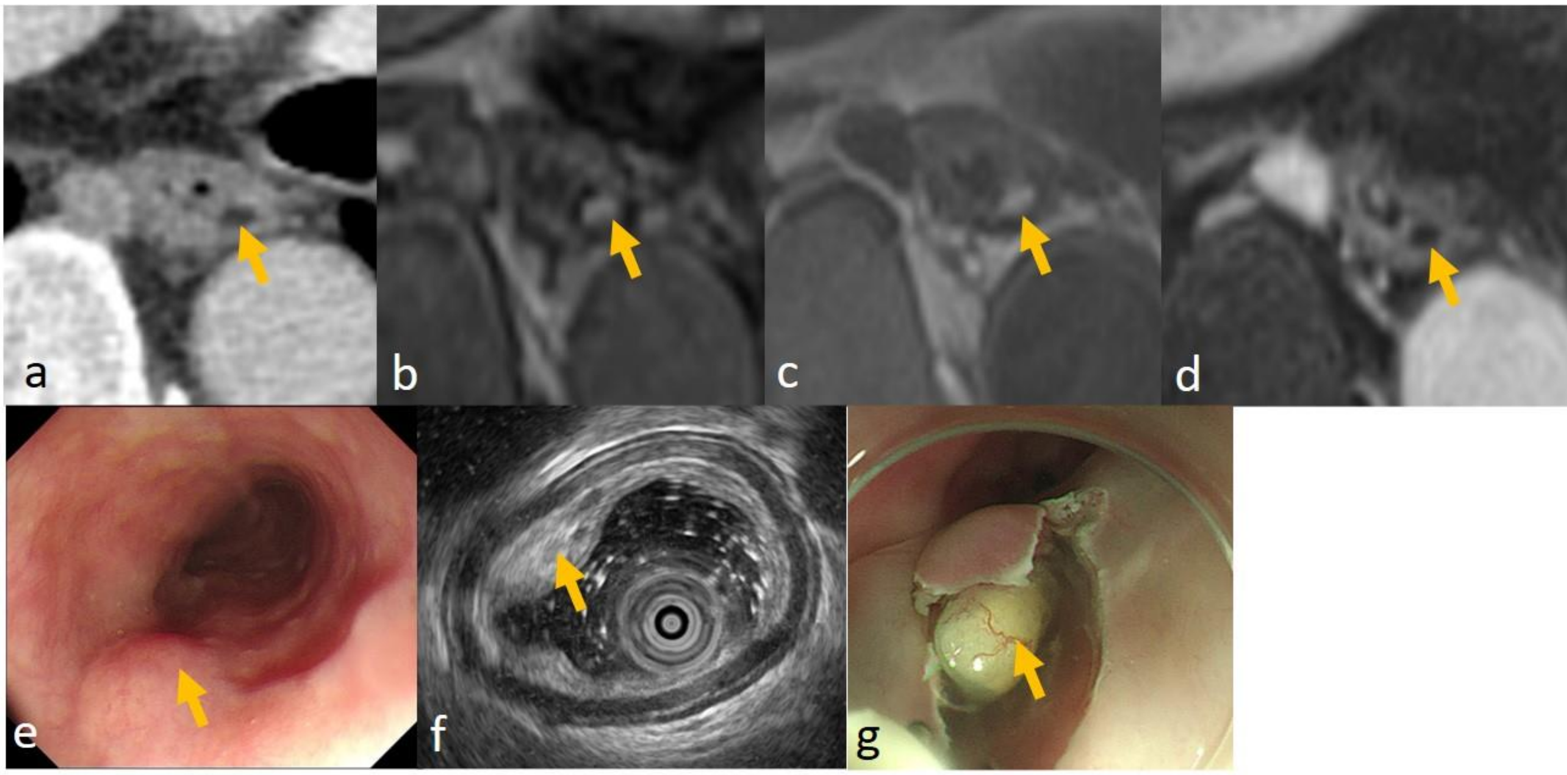

Figure S6. Esophageal hemangioma in a 58-year-old woman. The lesion (white arrow) shows similar enhancement to mucosa (blue arrow) with strong homogenous enhancing stalk (yellow arrow) on contrast-enhanced T1-weighted image (a, b). The mass is moderate hyperintense on T2-weighted image (c). CT shows a poorly defined enhancing mass (white arrow) after contrast injection without definite pedicle (d). PET/CT shows the mass with SUV value of 7.1 (e). Oesophagoscope (f) and endoscopic ultrasonography (g) shows the lesion. Intraoperative photography showed the stalk of the mass (h). It was confirmed to be granulation tissue-type hemangioma histopathologically.

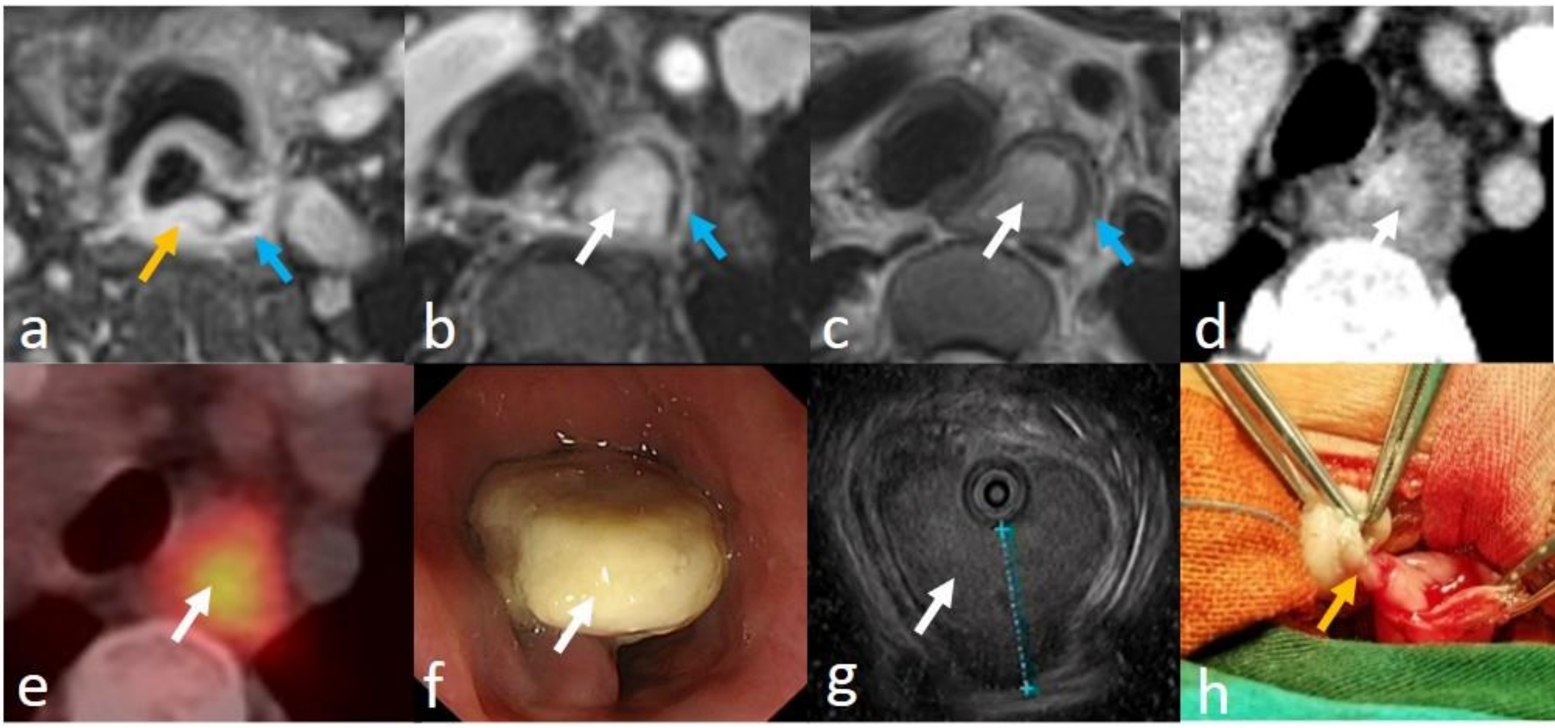

Figure S7. Fungal infected cyst images in a 23-year-old woman. Lesion shows a submucosal homogenous unenhanced mass (white arrow) after contrast injection on CT image (a), isointensity on T1-weighted image (b), moderate heterogeneous hyperintensity, which is slightly higher than mucosa (yellow arrow) on T2-weighted image (c), no diffusion restriction (d, e), and homogenous unenhanced mass after contrast injection on T1-weighted image (f). Oesophagoscope (g) and endoscopic ultrasonography (h) shows this submucous protrusion lesion. Fungal filaments can be seen on the esophageal lump puncture fluid base (H&E-stained section, i), which shows fungal mycelium (blue arrow) within the stacked squamous epithelium (purple arrow).

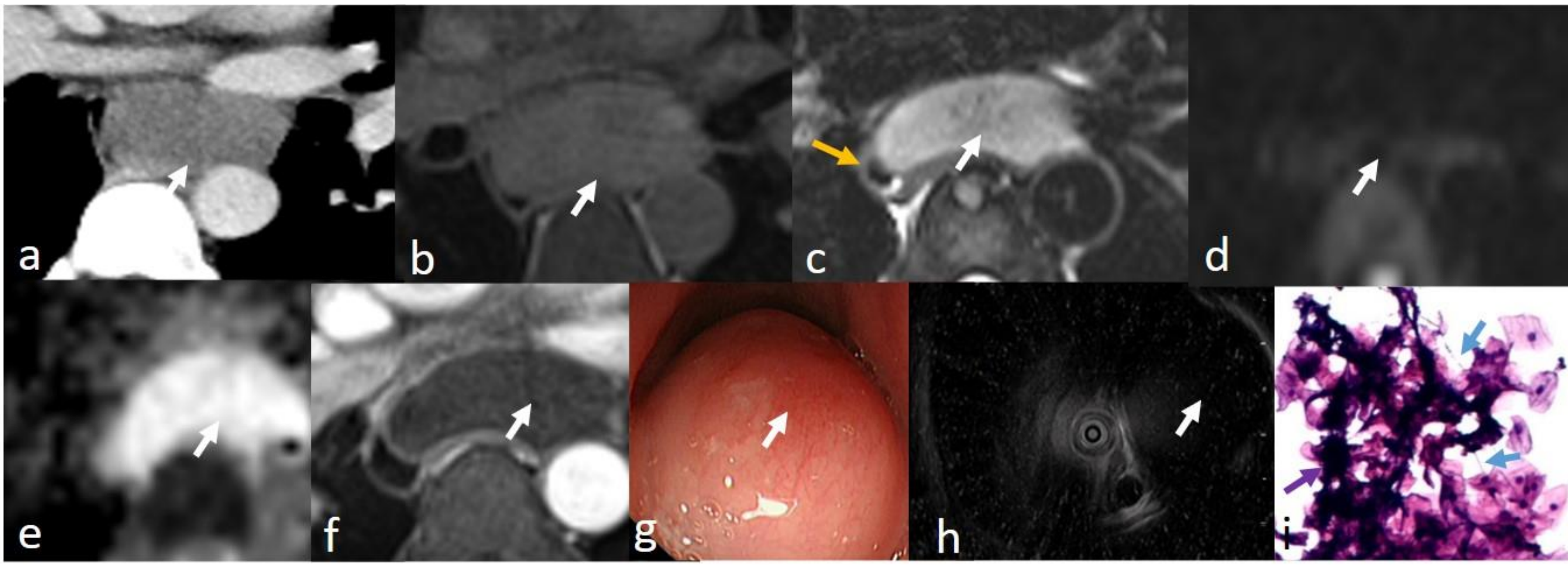

Supplement: Supplementary file 1 — ELECTRONIC SUPPLEMENTARY MATERIAL [file 13244_2024_1754_MOESM1_ESM.pdf]
